# Longitudinal inspiratory capacity changes in chronic obstructive pulmonary disease

**DOI:** 10.1186/1465-9921-13-66

**Published:** 2012-08-06

**Authors:** Bartolome R Celli, Marc Decramer, Theodore Lystig, Steven Kesten, Donald P Tashkin

**Affiliations:** 1 Pulmonary Division, Brigham and Women’s Hospital, Boston, MA, USA; 2 Respiratory Division, University of Leuven, Leuven, Belgium; 3 Respiratory Department, Boehringer Ingelheim, Pharmaceuticals Inc, Ridgefield, Connecticut, USA; 4 Department of Medicine, David Geffen School of Medicine at UCLA, Los Angeles, CA, USA

**Keywords:** COPD, Inspiratory Capacity, Tiotropium

## Abstract

**Background:**

The changes in inspiratory capacity (IC) over time in chronic obstructive pulmonary disease (COPD) patients are unknown. The Understanding Potential Long-term Impacts on Function with Tiotropium (UPLIFT®) trial included IC measurements.

**Methods:**

IC analysis from UPLIFT® (N = 5992) was performed at 1 and 6 months, and every 6 months through 4 years. Annualized rate of decline in pre- and post-bronchodilator IC and mean differences at each time point were analyzed by mixed-effects models. The relationships between baseline IC and exacerbation rate and mortality were explored using Cox regression analysis.

**Results:**

Baseline characteristics: age, 65 years; 75% men; post-bronchodilator forced expiratory volume in 1 second, 1.32 L (48% predicted); pre- and post-bronchodilator IC, 2.03 and 2.33 L. Mean IC rate of decline (mL/year) was 34 ± 2 (1.7% of baseline) and 50 ± 3 (2.1% of baseline) pre- and post-bronchodilator, respectively, without significant between-group differences. Morning pre-bronchodilator (trough) IC improved with tiotropium versus placebo: 124 mL (1 month), 103 mL (1 year), 107 mL (2 years), 98 mL (3 years), and 97 mL (4 years) (all p < 0.001). Post-bronchodilator improvements were similar between treatment groups. Lower baseline IC values were associated with reduced time to first exacerbation. For the lowest quartile (n = 1413) the values in months were 14.3 (11.7–17.0) for tiotropium and 10.3 (8.8–11.7) for controls (p < 0.01).

**Conclusion:**

IC declines from approximately 34 to 50 mL/year in patients with stage II to IV COPD. Tiotropium treatment does not change the IC decline rate but provides 24-hour improvements in IC sustained over the long term. Trough IC differences suggest that tiotropium provides sustained decrease in end-expiratory lung volume.

## Background

It has been increasingly recognized that lung volumes have an independent important association with symptom limitations and outcomes, including survival, in patients with chronic obstructive pulmonary disease (COPD) [1-5]. As the disease progresses, air trapping and hyperinflation develop, which worsen during physical activity and exacerbations. Increases in inspiratory capacity (IC) are positively associated with exercise duration and decreased breathlessness during activity [2-4]. The association appears to be stronger than the association of forced expiratory volume in 1 second (FEV_1_) with these outcomes and therefore represents an important surrogate of the functional impact of COPD on patient-reported outcomes.

While the annualized rate of decline in FEV_1_ has been well studied, there is no information regarding the rate of change in IC in patients with COPD [6-14]. Few studies have incorporated long-term evaluations of lung function beyond FEV_1_ and forced vital capacity (FVC) and none have reported on the rate of decline in IC.

The Understanding Potential Long-term Impacts on Function with Tiotropium (UPLIFT®) trial is a 4-year, randomized, double-blind, placebo-controlled trial of tiotropium 18 μg daily in patients with COPD who were permitted to use all respiratory medications throughout the trial, other than inhaled anticholinergics [13]. In UPLIFT®, the IC was measured during the pre- and post-bronchodilator slow vital capacity (SVC) maneuvers using a standardized spirometric technique. We conducted the following analyses on the UPLIFT® data: first, we investigated changes in IC over time in patients with COPD; second, we evaluated the effect of tiotropium (compared with control) on trough and post-bronchodilator IC over 4 years; third, we determined the changes in SVC and forced expiratory volume in 6 seconds (FEV_6_); finally, we explored the relationship between IC at baseline and time to first exacerbation, as well as mortality.

## Methods

### Study design

The study design details from the UPLIFT® trial have been published previously [12,13]. UPLIFT® was a randomized, double-blind, placebo-controlled clinical trial evaluating tiotropium 18 μg daily over 4 years in patients with COPD. The rates of decline in pre- and post-bronchodilator FEV_1_ were the primary endpoints. Absolute values for spirometry over 4 years, exacerbations, health-related quality of life (according to St George’s Respiratory Questionnaire [SGRQ]) [15]; only baseline data reported here), and mortality variables were secondary outcomes. All maintenance respiratory medications, except for inhaled anticholinergics, were permitted. The study had Institutional Review Board approval and all patients gave written informed consent. The study was conducted in accordance with the Declaration of Helsinki.

### Patients

Patients were included based on a clinical diagnosis of COPD, post-bronchodilator FEV_1_ ≤ 70% of predicted, FEV_1_/FVC ≤ 0.70, aged ≥ 40 years, and a smoking history of ≥ 10 pack-years. The major exclusion criteria were as follows: history of asthma, COPD exacerbation within 4 weeks of screening, prior pulmonary resection, and supplemental oxygen use > 12 hours per day.

### Lung function outcomes

Spirometry was performed according to American Thoracic Society (ATS) criteria [16] at baseline, at 30 days’ post-randomization, at every 6 months’ post-randomization, and at 30 days following the last dose of study medication. Spirometry was conducted before and after short-acting bronchodilators (ipratropium 80 μg, then albuterol 400 μg 60 minutes later). Following randomization, study drug was administered immediately prior to short-acting bronchodilators.

Spirometry was performed with the same equipment at each site using trial-specific customized software. Results were transmitted for centralized quality assurance assessment (Quantum Research Inc, Louisville, Colorado, USA). SVC by slow exhalation maneuver was performed first, followed by the FVC maneuver. Both maneuvers were performed three times with up to five forced expiratory maneuvers permitted in order to obtain three acceptable efforts. The highest acceptable FEV_1_ and the highest FVC obtained on any of the three acceptable efforts were recorded.

IC was measured in maneuvers used to generate the SVC. All investigators were trained to collect IC and SVC measurements, and the technique used was standard and supervised at all centers. A computerized algorithm helped stabilize the end-expiratory signal and prompted technicians to perform IC and SVC maneuvers that met ATS standards. All patients were sitting and continuous recording was made of the tidal volume. When patients achieved a tidal volume signal with stable end-expiratory volume, they were instructed to inspire as deeply as possible until no more air could be inhaled. At that point, the patient performed the SVC maneuver until residual volume was reached. The maneuvers were repeated after 5 minutes’ rest until three appropriate, reproducible tracings were obtained. The best IC and SVC were used for analyses.

### Exacerbations, health status, and mortality

Exacerbation data and adverse events were collected at each clinic visit. For the purpose of this analysis, exacerbations were defined by episodes of shortness of breath and change in the sputum that merited a course of corticosteroids or antibiotics (or both) and/or required a visit to an emergency room or admission to hospital. The SGRQ was administered at baseline and every 6 months. Mortality was determined up to Day 1470 after randomization [13].

### Statistical methods

Data from all randomized patients with acceptable pre- and post-bronchodilator measurements at baseline were included in this analysis. A mixed-effects model with repeated measurements analysis of variance was utilized to determine the rate of change in spirometry variables. Data sets were restricted to patients with ≥ 3 post-randomization spirometry test sets for calculation of annualized rate of decline in FEV_1_, FVC, and SVC, and to patients with ≥ 1 post-randomization spirometry test set for calculation of annualized rate of decline in IC and FEV_6_. IC at baseline was divided into approximate quartiles using the cut points of 1.5, 2.0, and 2.5 L. Hazard ratios for the risk of an exacerbation and for mortality were calculated using Cox regression.

## Results

### Study population

The demographics of the UPLIFT® population have been previously described and are displayed in Table 1[13]. Using the 2010 grading of airflow obstruction as characterized by the Global Initiative for Chronic Obstructive Lung Disease (GOLD) [1], the mean post-bronchodilator FEV_1_ was 48 % predicted (Table 2) with 46 %, 44 %, and 9 % stage II, III, and IV disease, respectively. Evaluable measurements of IC at baseline were available for 5992 patients.

**Table 1:** Baseline characteristics of patients in the tiotropium and control groups

**Characteristic**	**Tiotropium (n = 2986)**	**Control (n = 3006)**
Male, %	75.4	73.9
Age, years^a^	64.5 ± 8.4	64.5 ± 8.5
Body mass index^a^	26.0 ± 5.1	25.9 ± 5.1
Smoking status		
Current smoker, %	29.3	29.9
Smoking history, pack-years^a^	49.0 ± 28.0	48.4 ± 27.9
SGRQ total score, units^a^	45.7 ± 17.0	46.0 ± 17.2
Respiratory medications, %		
Short-acting inhaled anticholinergics^b^	44.9	44.1
Long-acting inhaled anticholinergics	2.0	1.6
Short-acting inhaled β_2_-agonists^b^	68.5	68.1
Long-acting inhaled β_2_-agonists^b^	60.1	60.1
Inhaled corticosteroids^b^	61.6	61.9
Oral steroids	8.4	8.3
Theophylline compounds	28.4	28.5
Mucolytics	7.4	6.9
Leukotriene receptor antagonists	3.3	3.1
Supplemental oxygen	2.3	1.9

^a^Mean ± SD. ^b^Used alone or as a fixed combination.

Abbreviation: SGRQ, St George’s Respiratory Questionnaire.

**Table 2:** Baseline spirometry (mean ± SD) of patients in the tiotropium and control groups

	**Tiotropium (n = 2986)**	**Control (n = 3006)**
Pre-bronchodilator		
FEV_1_, L	1.10 ± 0.40	1.09 ± 0.40
FEV_1_, % predicted	39.5 ± 12.0	39.3 ± 11.9
FVC, L	2.63 ± 0.81	2.63 ± 0.83
FEV_1_/FVC	42.4 ± 10.5	42.1 ± 10.5
SVC	2.80 ± 82	2.80 ± 83
IC	2.04 ± 70	2.03 ± 70
FEV_6_	2.11 ± 62	2.11 ± 62
Post-bronchodilator		
FEV_1_, L	1.33 ± 0.44	1.32 ± 0.44
FEV_1_, % predicted	47.7 ± 12.7	47.4 ± 12.6
FVC, L	3.09 ± 0.86	3.09 ± 0.90
FEV_1_/FVC	43.6 ± 10.8	43.3 ± 10.7
SVC	3.21 ± 88	3.20 ± 90
IC	2.35 ± 75	2.32 ± 77
FEV_6_	2.49 ± 65	2.48 ± 66

Abbreviations: FEV_1_, forced expiratory volume in 1 second; FEV_6_, forced expiratory volume in 6 seconds; FVC, forced vital capacity; IC, inspiratory capacity; SD, standard deviation; SVC, slow vital capacity.

### IC and SVC

**Figure 1:**
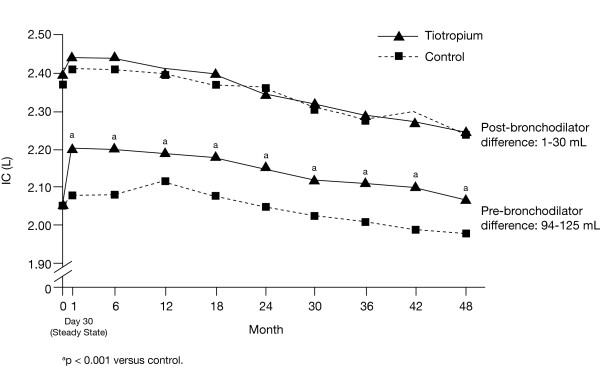
**Estimated mean pre- and post-bronchodilator IC in the tiotropium and control groups through 4 years.** Abbreviations: IC, inspiratory capacity.

**Figure 2:**
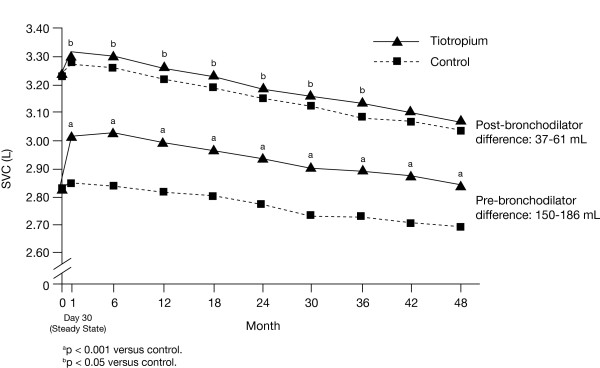
**Estimated mean pre- and post-bronchodilator SVC in the tiotropium and control groups through 4 years.** Abbreviations: SVC, slow vital capacity.

**Figure 3:**
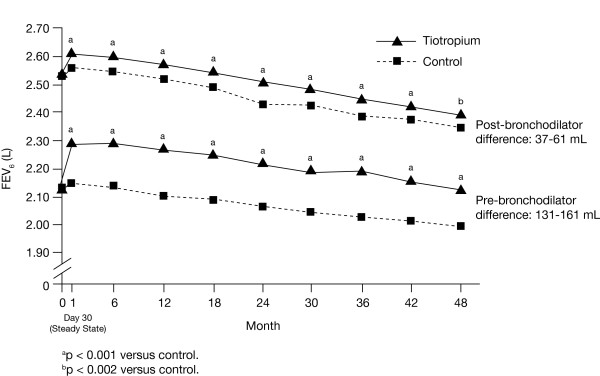
**Estimated mean pre- and post-bronchodilator FEV**_**6**_**in the tiotropium and control groups through 4 years.** Abbreviations: FEV_6_, forced vital capacity in 6 seconds.

Improvements in morning pre-bronchodilator IC were observed throughout the trial, with the differences between tiotropium and control ranging from 94 to 125 mL (p < 0.001 at all time points) (Figure 1). Significant improvements were also observed for pre-bronchodilator SVC (between-treatment differences between 150 and 186 mL, p < 0.001 at all time points) (Figure 2), and FEV_6_ (between-treatment differences between 131 and 161 mL, p < 0.001 at all time points) (Figure 3). Differences observed for FVC were between 170 and 204 mL (p < 0.001) [13].

Pre-bronchodilator IC declined from 32 to 36 mL/year while post-bronchodilator IC declined from 47 to 52 mL/year, with no differences between treatment groups (Table 3). The corresponding rates of decline for SVC were numerically larger, with pre-bronchodilator values between 41 and 47 mL/year and post-bronchodilator values between 65 and 66 mL/year; corresponding values for FEV_6_ were 40–42 mL/year and 57 mL/year, with no between-treatment differences (Table 3).

**Table 3:** **Annual rates of decline in pre-/post-bronchodilator IC, FEV**_**6**_**, and SVC over 4 years**^**a**^

	**Tiotropium (mL/year)**		**Control (mL/year)**		Δ **Tiotropium–control**	**p-value**
	**N**	**Mean (SE)**	**N**	**Mean (SE)**	**Mean (SE)**	
Pre-bronchodilator	2910	36 (3)	2864	32 (3)	5 (5)	0.30
Post-bronchodilator	2905	52 (4)	2853	47 (4)	6 (5)	0.28
Pre-bronchodilator	2910	42 (2)	2864	40 (2)	2 (3)	0.39
Post-bronchodilator	2905	57 (2)	2853	57 (2)	−0 (3)	0.91
Pre-bronchodilator	2531	47 (3)	2374	41 (3)	6 (4)	0.11
Post-bronchodilator	2527	66 (3)	2383	65 (3)	1 (4)	0.79

^a^Data from SVC previously published [13].

Abbreviations: FEV_6_, forced expiratory volume in 6 seconds; IC, inspiratory capacity; SE, standard error; SVC, slow vital capacity.

The mean rate of decline in pre-bronchodilator IC was similar for patients at GOLD stage II (placebo group, 33 mL/year; tiotropium group, 37 mL/year), stage III (34 and 37 mL/year, respectively), and stage IV (20 and 34 mL/year), but increased with the severity of airflow obstruction for post-bronchodilator IC (GOLD stage II, 41 and 44 mL/year; stage III, 48 and 56 mL/year; stage IV, 57 and 63 mL/year for the placebo and tiotropium groups, respectively).

Table 4 illustrates the treatment group pre-bronchodilator differences at 1, 2, 3, and 4 years for IC, SVC, FEV_6_, and FVC. Treatment group differences in IC appear fairly stable over 4 years. SVC, FEV_6_, and FVC showed the largest decline in between-group differences from Year 3 to Year 4, with minor declines in Year 4.

**Table 4:** **Mean between-treatment differences up to 4 years in pre-bronchodilator IC, SVC, FEV**_**6**_**, and FVC**

	**1 Year**	**2 Years**	**3 Years**	**4 Years**
IC (SE)	103 (17)	107 (18)	98 (19)	97 (20)
SVC (SE)	176 (13)	167 (14)	166 (15)	150 (16)
FEV_6_ (SE)	159 (9)	149 (10)	161 (11)	131 (12)
FVC^a^ (SE)	198 (13)	189 (14)	200 (15)	170 (16)

^a^Data from FVC previously published [13].

Abbreviations: FEV_6_, forced expiratory volume in 6 seconds; FVC, forced vital capacity; IC, inspiratory capacity; SE, standard error; SVC, slow vital capacity.

Associations among values were determined at individual time points. The correlations between IC and FEV_1_ varied between 0.51 and 0.60 (pre-bronchodilator) and between 0.45 and 0.54 (post-bronchodilator). The correlations between IC and FVC varied between 0.57 and 0.66 (pre-bronchodilator) and between 0.55 and 0.63 (post-bronchodilator). The correlations between IC and SVC varied between 0.63 and 0.74 (pre-bronchodilator) and between 0.61 and 0.70 (post-bronchodilator).

### Relationship of baseline IC to exacerbations and mortality

The mean (range) of pre-bronchodilator baseline IC by quartiles were: Q1, 1.15 (0.45–1.50); Q2, 1.76 (1.51–2.00); Q3, 2.25 (2.01–2.50); Q4, 2.95 (2.51–4.00). Lower quartiles of IC were associated with a faster time to first exacerbation within each treatment group (Table 5). Tiotropium was associated with a lower risk for an exacerbation within each quartile, although the upper limit of the 95% confidence interval (CI) exceeded one for the third quartile. Lower quartiles of IC were associated with a higher risk for a fatal event within each treatment group (Table 6). While the risk for a fatal event was reduced with tiotropium in each quartile, in all cases, the 95% CI included one, likely influenced by the relatively low number of events within each quartile (Table 6).

**Table 5:** Time to first exacerbation (mean months and HRs) (95 % CI) according to baseline IC quartiles

**IC quartile**	**Tiotropium (n = 2855)**	**Control (n = 2881)**	**HR (95 % CI)**
Q1 (n = 1413)	14.3 (11.7-17.0)	10.3 (8.8-11.7)	0.81 (0.72-0.92)
Q2 (n = 1427)	14.7 (12.6-17.6)	10.5 (8.7-12.2)	0.83 (0.73-0.94)
Q3 (n = 1450)	16.8 (13.6-19.8)	14.8 (12.2-17.2)	0.95 (0.83-1.07)
Q4 (n = 1446)	20.4 (17.3-23.4)	15.8 (12.8-18.4)	0.86 (0.75-0.97)

Abbreviations: CI, confidence interval; HR, hazard ratio; IC, inspiratory capacity.

**Table 6:** All-cause mortality (n [%]) (95 % CI) until Day 1470 according to baseline IC quartiles

**IC quartile**	**Tiotropium (n = 2855)**	**Control (n = 2881)**	**HR (95 % CI)**
Q1	141/714 (19.7 %)	158/699 (22.6 %)	0.85 (0.68-1.07)
Q2	115/669 (17.2 %)	136/758 (17.9 %)	0.95 (0.74-1.22)
Q3	94/735 (12.8 %)	104/715 (14.5 %)	0.86 (0.65-1.13)
Q4	64/737 (8.7 %)	78/709 (11.0 %)	0.79 (0.56-1.09)

Abbreviations: CI, confidence interval; HR, hazard ratio; IC, inspiratory capacity.

## Discussion

This analysis from the UPLIFT® study offered some novel findings. Firstly, the pre-bronchodilator IC declined from 32 to 52 mL/year in patients with COPD included in the 4-year trial. Secondly, the rate of decline was similar in patients taking tiotropium compared with patients taking control plus regular medications, but the morning IC (trough) was significantly higher in patients taking tiotropium throughout the study. Thirdly, the changes in IC were mirrored by the changes in SVC. Fourthly, lower values of IC at baseline were associated with higher rates of exacerbations and death.

A series of studies has shown that the end-expiratory lung volume (EELV) is an important determinant of exercise limitation and the rate of development of dyspnea during exercise, as well as a good predictor of survival [2-5]. Improvement in clinically relevant outcomes such as increases in exercise endurance time and decreases in dyspnea have been related more strongly to changes in EELV than to changes in FEV_1_[2-4,17,18].

Furthermore, data from the National Emphysema Therapy Trial (NETT) have shown improvements in survival in selected patients who underwent surgical lung volume reduction compared with patients randomized to medical therapy [19]. Taken together, these studies strongly support the concept that EELV is an important determinant of functional capacity and outcome in patients with COPD.

The IC has been shown to reflect EELV and can be measured at rest using a spirometer and during exercise using relatively simple maneuvers [17,20,21]. Until now, there has been no description of the behavior of IC over long periods of time and of the effect of pharmacologic therapy on those changes. The measurements of IC and SVC at baseline and at each of the subsequent visits were incorporated as part of the standardized spirometry equipment and software. Such data were envisioned to help close gaps in knowledge in this important area.

On average, the IC declines over time at a rate that is similar to the decline in FEV_1_. This supports the clinical observation that, as disease progresses, many patients with COPD will develop hyperinflation. Since the publication of the study by Fletcher and Peto, it has been classically stated that the rate of decline of FEV_1_ in patients with COPD oscillates around 60 mL/year [6]. Evidence from the UPLIFT® and Towards a Revolution in COPD Health (TORCH) trials showed that the actual rate of decline is lower, being on average close to 40 mL/year [13,14]. Furthermore, recent analysis of the spirometric records of the second Framingham cohort by Kohansal *et al.*[22] showed that the normal rate of decline is approximately 19 mL/year and 30–40 mL for smokers in that same population. It seems as if the rate of decline, at least for FEV_1_, has decreased over the time that has elapsed since the report by Fletcher and Peto [6]. It cannot be determined whether this is due to changes in therapy, the environment, or the anthropometric constitution of populations, but the consequence is that a ceiling effect may have been reached, beyond which it will be very difficult to change the rate of decline any further, other than through smoking cessation in patients who continue to smoke. However, it is theoretically possible to attempt to erase the additional relatively small decline that patients with COPD have compared with non-smoker controls. Interestingly, we found that the rate of decline of post-bronchodilator IC was greater in patients with more severe airflow obstruction (GOLD stage IV, compared with stages III or II). This finding differs from the pattern observed for FEV_1_ in the UPLIFT® and TORCH studies, in which there was a greater decline over time in patients with milder airflow limitation [13,14].

Even though the rate of decline of IC in UPLIFT® was similar in patients with tiotropium when compared with controls, trough IC was larger by approximately 100 mL in the tiotropium group. These results are consistent with shorter-term studies with tiotropium [17,18,20,23]. Although we did not measure total lung capacity (TLC), evidence from shorter-term studies suggests that this may decrease after bronchodilators, but the decrease is relatively small, so that the ratio of IC/TLC is likely to have improved in the patients taking tiotropium compared with those on control [17,20]. The 4-year UPLIFT® trial substantiated previous reports from trials up to 1 year in duration, demonstrating that tiotropium improves exacerbation rates and health-related quality of life. In addition, the UPLIFT® trial showed that tiotropium could improve survival [13,24]. Such impacts on the course of COPD are likely to be related to, or at least influenced by, the change in IC. The data are insufficient to prove a cause-and-effect relationship, but recent evidence describing a relationship between hyperinflation and cardiac function, more specifically left ventricular stroke volume, supports this possible mechanism [25]. Indeed, the data are consistent with a beneficial effect on cardiac morbidity suggested by the results of the UPLIFT® and TORCH trials [13,26]. More studies are needed to explore the impact of changes in lung function on cardiac physiology.

That the IC findings are real is supported by the measurement of two other volume variables, the FEV_6_ and, more importantly, the SVC. It is known that, in patients with COPD, there is a difference in favor of the vital capacity between the value obtained with the slow maneuver and the value obtained during the forced maneuver (i.e. FVC). The SVC in this study was on average 100 mL larger than the FVC in the same patients, confirming in a large population what was already known from smaller physiologic studies. What had not been explored was the relationship between IC and SVC. In the current analysis, there was a strong association between IC and SVC, suggesting that one could potentially be used as a surrogate marker of the other.

Since we measured both FEV_1_ and IC in UPLIFT®, we sought to determine if both variables changed in tandem, in the same patients and in the same direction, and whether both variables had a similar prognostic value for the patient-reported outcomes studied in UPLIFT®. In relation to the first objective, we observed a close association between FEV_1_ change and IC change, although the associations between IC and either SVC or FVC appeared to be stronger. Furthermore, the changes occurred in the same direction in the same patients, thereby making one variable a potential surrogate marker of the other. Given the similar strength of their association, it seems appropriate to use the FEV_1_ as the single most stable physiologic variable for the study of these outcomes. UPLIFT® was not designed to answer the question of the association of IC with exercise capacity and, therefore, this remains to be determined.

There were some limitations to this study. It is possible that the longitudinal IC changes may have been affected by the rate of discontinuations in UPLIFT®, which was approximately 40% overall [13]. However, similar discontinuation rates have been reported in other large-scale trials of respiratory maintenance therapies in COPD [12]. There was no centralized review of the quality of the IC, although this was undertaken for all slow and forced expiratory maneuvers that by definition included the IC. In addition, the large number of patients and the large number of maneuvers incorporated reduces the likelihood of variability and randomness as an explanation for the results. There are no validated international predicted values for IC, FEV_6_, and SVC, and no information about the minimally clinical important difference, so we had to limit the analyses to changes in absolute values of these variables. It is unlikely that expressing these variables as a change in their predicted values or, in the case of IC, as a change in its ratio to TLC would have improved the strength of the analysis or its conclusions.

## Conclusions

In summary, in patients with GOLD stage II to IV COPD included in the UPLIFT® study, mean pre- and post-bronchodilator IC declined from 32 to 36 mL per year and from 47 to 52 mL per year, respectively. Treatment with tiotropium did not change the rate of decline of IC over time but provided 24-hour improvements sustained over the long term. The difference in trough IC between tiotropium and control suggests that tiotropium resulted in a sustained decrease in EELV. Both IC and SVC provided information that related to clinical outcomes, but the close association between changes in these variables and changes in FEV_1_ indicates that the FEV_1_ remains a relevant physiological variable of use in the study of patients with COPD. Long-term studies of the changes in lung volumes and their relation to exercise performance and mortality should be undertaken to clarify the role of IC and other lung volumes in the routine management of patients with COPD.

## Abbreviations

CI, confidence interval; COPD, chronic obstructive pulmonary disease; EELV, end-expiratory lung volume; FEV1, forced expiratory volume in 1 second; FEV6, forced expiratory volume in 6 seconds; FVC, forced vital capacity; GOLD, Global Initiative for Chronic Obstructive Lung Disease; IC, inspiratory capacity; NETT, National Emphysema Therapy Trial; SGRQ, St George’s Respiratory Questionnaire; SVC, slow vital capacity; TLC, total lung capacity; TORCH, Towards a Revolution in COPD Health; UPLIFT®, Understanding potential long-term impacts on function with tiotropium.

## Competing interests

Steven Kesten, MD, and Theodore Lystig, PhD, were employees of Boehringer Ingelheim at the time the study was conducted. Bartolome R Celli, MD, Marc Decramer, MD, and Donald P Tashkin, MD, have no conflicts of interest to disclose.

## Authors’ contributions

All authors had access to the data and had a role in writing the manuscript.
